# Applying a 6 DoF Robotic Arm and Digital Twin to Automate Fan-Blade Reconditioning for Aerospace Maintenance, Repair, and Overhaul

**DOI:** 10.3390/s20164637

**Published:** 2020-08-18

**Authors:** John Oyekan, Michael Farnsworth, Windo Hutabarat, David Miller, Ashutosh Tiwari

**Affiliations:** The Department of Automatic Control and Systems Engineering (ACSE), Faculty of Engineering, The University of Sheffield, Sheffield S10 2TN, UK; m.farnsworth@sheffield.ac.uk (M.F.); w.hutabarat@sheffield.ac.uk (W.H.); d.b.miller@sheffield.ac.uk (D.M.); a.tiwari@sheffield.ac.uk (A.T.)

**Keywords:** Industry 4.0, automation, digital twin, analytics, computer vision

## Abstract

The UK is home to several major air commercial and transport hubs. As a result, there is a high demand for Maintenance, Repair, and Overhaul (MRO) services to ensure that fleets of aircraft are in airworthy conditions. MRO services currently involve heavy manual labor. This creates bottlenecks, low repeatability, and low productivity. Presented in this paper is an investigation to create an automation cell for the fan-blade reconditioning component of MRO. The design and prototype of the automation cell is presented. Furthermore, a digital twin of the grinding process is developed and used as a tool to explore the required grinding force parameters needed to effectively remove surface material. An integration of a 6-DoF industrial robot with an end-effector grinder and a computer vision system was undertaken. The computer vision system was used for the digitization of the fan-blade surface as well as tracking and guidance of material removal. Our findings reveal that our proposed system can perform material removal, track the state of the fan blade during the reconditioning process and do so within a closed-loop automated robotic work cell.

## 1. Introduction

The demand for manufacturing systems that enable the Maintenance, Repair, and Overhaul (MRO) of aircraft is increasing. Manufacturing Systems for MRO are essential in ensuring that an airline’s fleet of aircraft are always in top conditions of airworthiness for transportation of goods and people [1]. MRO is important because the commercial air transport MRO market is growing. Total MRO spend is expected to rise to £116B by 2029 from the current £81.9B in 2019 [2]. Furthermore, MRO ensures that the earth’s resources are used in a sustainable manner.

With advances in material research, most of the new generation engines are increasingly being made with composite materials to ensure strong structures, lighter weights, and lower fuel consumption. For example, Pratt & Whitney have recently developed and produce an engine with reduced fuel burn, noise, and maintenance costs. Also, it provides a new level of performance and operational efficiency for airline customers [3]. Due to an increasing demand for these engines, an extensive network of MRO facilities is required to reduce the time aircraft spend in maintenance hangars. One of the parts of the engine that requires continuous maintenance is the Turbofan engine’s fan blade.

These key component’s surfaces are sometimes damaged during operation. Damages include cracks in the surface coat, adhesive joint failures, delamination, splitting along fibers to mention a few [4]. During the MRO process, each fan blade must be brought back to original specification. This involves both repair and a process of grinding to strip the top two layers of protective coating (epoxy primer and polyurethane coating) of the fan blade. Currently, this grinding process is done manually. The manual process is lengthy and often subjects workers to Musculoskeletal strains. Furthermore, the environment in which grinding takes place requires the use of protective clothing, breathing apparatus, and limited operation time due to the nature of the orbital sanders used to grind away material [5,6].

Since the manual grinding process is a dull, dirty, and dangerous task, it meets the criteria for automation. The use of industrial robotic solutions coupled with recent advances in sensing, digitization, and digital twins provide a way forward for future automated MRO systems.

The development of such automated systems, requires an understanding of the current reconditioning process in order to understand the visual sensory processes used by engineers, the knowledge used by the engineers to determine the grinding force to apply to the blade as well as the fine-tuning used by the engineer during grinding [5,6,7]. Such understanding will provide knowledge of sensor interrogation of the material being reconditioned, automation as well as output in the form of grinding forces.

Grinding is a complex process and the geometry of the abrasive surface/pad among other factors controls the surface roughness [8]. Due to the short contact time and small contact zone, the grinding process is hard to observe directly. As a result, understanding the cutting forces and chip formation processes from a mathematical perspective can shed light into what happens during grinding and how the process can be controlled to achieve the desired effect [8].

Compared to wet grinding operations, dry grinding conditions are eco-friendly. They enable the abrasive material to be collected and recycled. They are also considerably cheaper in that lubricants are not used. However, it increases the effect of thermal damage to the surfaces [9]. As a result, the process parameters must be controlled closely to achieve the desired surface roughness while remaining within process constraints.

In this work, automated dry grinding is investigated. The first step in applying restorative grinding forces to a fan blade is the quantification of the defect in a region. This should ideally be done non-destructively to preserve the integrity of the fan blade. Towards this, non-destructive tests (NDT) are often conducted. NDT methods are divided into non-contact and contact. Contact methods often require good contact between the sensor and tested composite surface to obtain reliable data. These methods include ultrasonic, eddy current, magnetic, electromagnetic, and penetrant tests. However, these contact methods are often slower than optical non-contact methods such as thermograph, holography or shearography [10,11]. As a result, in this work we focus on the use of optical techniques for inspection of blade surfaces. Even though damage assessment using external visual inspection techniques cannot identify the inner damages in the blades [4], visual inspection is currently used by workers to manually inspect the surfaces of the blade. They typically look for surface cracks, dents, areas damaged by lightning strikes, debonding among other damages when inspecting blades. Since these defects could be at the surface level, middle, or at the core, the magnitude of the damage determines the amount of repair regime they will apply to an area. The visual inspection task is conducted in the visible spectrum and as a result, we opt for RGB-D (cameras with the ability to sense Red, Green and Blue color spectrums as well as Depth) cameras to replicate the manual visual inspection task. The aim was to digitize this task and use it to inform the development of an automated rig for material removal of the fan blade during reconditioning.

The use of cameras and vision-based algorithms for inspecting automated processes has been investigated extensively in the literature. These include the use of various 3D object recognition algorithms [12,13,14], Convolutional Neural Networks for corrosion detection of regions [15,16] and the use of knowledge-based vision algorithms to identify regions of interest for closer inspection [17,18]. In a similar way as [17], we make use of the knowledge of the blade layer structure to develop a transition-based algorithm (discussed in Section 2) that enables us to assess and quantify the material state on the blade. The fan blade is composed of several material layers. However in this case study, we consider the first three top layers only: Composite (inner most layer); primer (middle layer); poly-ethylene-based coating (outer most layer).

After quantifying the defects, control software is required to develop a robust automated surface treatment system. Towards this, it is important to capture, understand the manual skills used in the process and then embed them in an automated process [19,20]. Various systems have been explored in the area of surface treatment such as the use of Computer Numerical Control (CNC) milling machines [21,22,23]. Also, software solutions that adapt to the shape of the geometry of the part being treated have been explored [20]. In these works, the variables explored have included the contact force between the part and the abrasive tool being used, feed rate, depth of cut, the speed of the rotating end-effector and its machining path. These process parameters affect the final surface quality. The authors of [24] presented a software tool that enables the automatic choosing of grinding tools in order to achieve different surface finishes while in [25], a robotic arm is used to generate and optimize a tool path so that the contact points between the tool tip and the workpiece are evenly distributed along the grinding path. Furthermore, in their work, the contact force’s direction and amplitude is measured with a force prediction algorithm and controlled with a force-position controller. As can be seen in all the work reviewed above, effective automation of a surface treatment process such as grinding requires a capturing and understanding of the manual process as well as the grinding forces at play at the interface of the rotating grinder and the surface [26,27]. This understanding also informs the development of grinding models and material removal prediction models that are useful for predicting the effects of process parameters. In MRO, this could improve the yield of MRO operations by reducing high workpiece rejection rates. It will also improve efficiency through faster reconditioning and ensuring right-first-time operations.

In the methodology section, we discuss how we digitized the manual visual inspection tasks performed by grinding operatives. We also discuss the forces an automated system might encounter during grinding. In Section 3, we present experiments and results. In Section 4, we discuss the results followed by conclusions in Section 5.

## 2. Methodology

**Figure 1:**
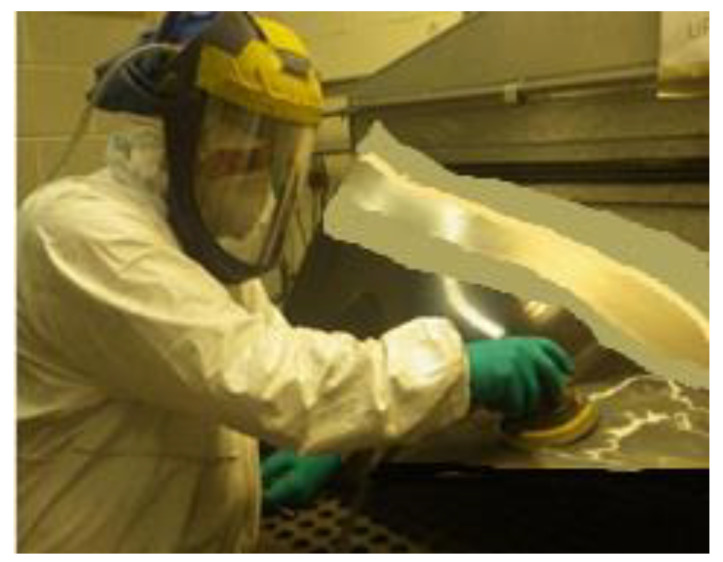
A worker performing manual grinding operation (We have smudge areas of the fan blade due to Intellectual Property reasons).

In this investigation, we worked with a major engine manufacturer and MRO provider towards developing an automated rig for grinding away the two top layers (poly-ethylene-based coating and primer) of a fan blade. Currently, grinding is done manually, and workers must wear uncomfortable protective clothing to protect from hazardous materials and particulates (Figure 1). Furthermore, they must adhere to several constraints during manual grinding which adds a mental burden on the operator. We shall discuss these constraints in Section 2.4.1.

**Figure 2:**
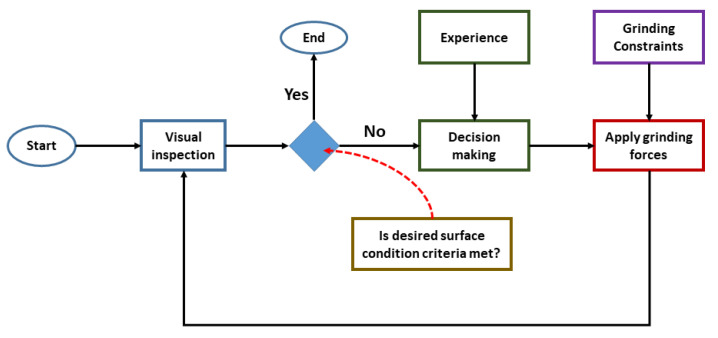
The manual stages involved in the manual grinding of fan blades during MRO.

**Figure 3:**
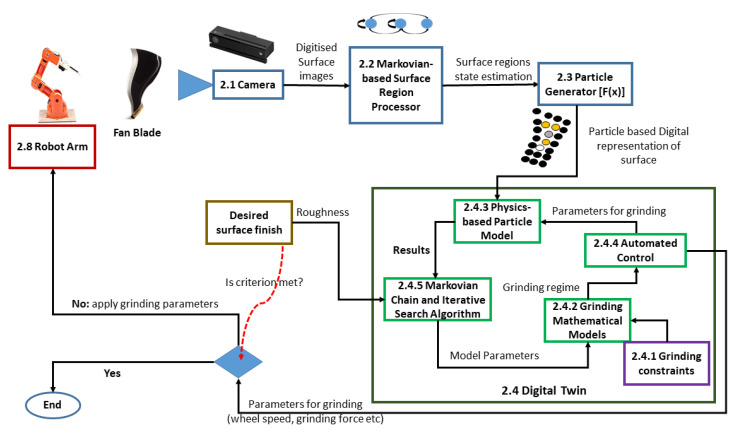
Showing our proposed automated architecture for MRO. The black dots above are used to depict the top polyurethane layer, the yellow or white dots the middle epoxy primer layer and the grey dots the innermost composite layer. The color scheme follows the format used in Figure 2. For example, purple depicts the grinding constraints for both human and the digital twin; Blue depicts an approach for digitizing a manual visual inspection. The desired surface finish module indicates the surface condition finish required. This indirectly provides the criterion for our architecture to decide whether to continue grinding or not. The numbers in the architecture’s module depict the section numbers in the manuscript.

In the current MRO process, the first step during reconditioning of fan blades is visual inspection (See Figure 2). This is used to determine the overall condition of the blade before manual grinding commences. Visual inspection also enables the operator to determine the grinding regime to apply to various regions of the fan blade. The results of the visual inspection is then used to make decisions on how much force to apply to the grinding head, speed of the grinding head across the surface among other grinding parameters (see Section 2.4.2). To achieve a certain surface finish, the experience of the operator becomes very important. Using Figure 2 as inspiration, we propose the architecture in Figure 3 towards achieving automation of the grinding process for MRO. The following subsections explain the various modules in the architecture.

### 2.1. Camera Based Visual Sensing Technique

Several possible vision-based sensing approaches where investigated. This included hyper-spectral cameras, lasers, surface tomography, and an ordinary RGB-D camera. Without incurring large costs, the laser scanning devices did not have the resolution required to determine the different depths between the layers especially during grinding. The resolution sort for was above 0.2 mm. Even though there are laser scanning devices that could achieve above this resolution, cost is a prohibitive factor for Small and Medium sized Enterprises (SMEs).

Furthermore, surface tomography approaches that used probes to determine surface depth were found to be slow and limited in their scanning size. Hyper-spectral cameras were investigated. However, the reflectivity and curvature of the blade coupled with lighting conditions made material depth measurement difficult. As a result, our focused shifted to tracking the state of material removal through the transition of the material color properties. Both the top polyurethane (black) and middle epoxy primer (white or yellow) have different color properties, making it possible to track the rate of material removal though the use of an RGB-D camera.

### 2.2. Markovian-Based Surface Region Processor

To apply the appropriate grinding forces to a blade, an algorithm capable of sensing the fan-blade surface state and characterizing it is required. This was because in some regions of the fan blade, the top layer of poly-ethylene could be damaged during operation and thus reveal the primer beneath. As a result, the grinding requirements for those regions will be different from the regions that have not been damaged.

**Figure 4:**
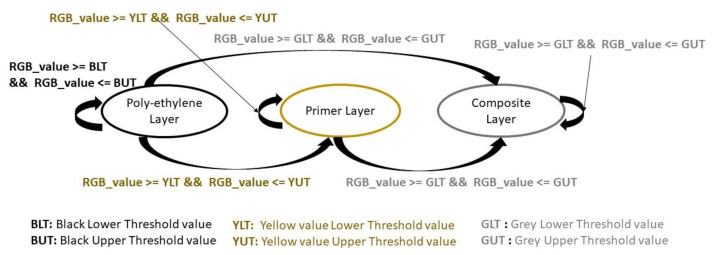
Showing the topmost layers of a fan blade and the transition between them. The rate of transition between the layers depend on the magnitude of the damage done to the fan blade, the time in service of the fan blade or the amount of grinding given to each layer.

We used a Markovian chain to model the condition of the fan-blade’s surface (Figure 4). Each state represents one of the topmost layers of the fan blade. The transition between the layers or the condition of the fan-blade’s surface is dependent on how long it has been in service, how much damage it has sustained as well as the amount of grinding it has been put through. We used Figure 4 to design algorithmic methods that replicate part of a human worker’s visual processes when inspecting a fan blade. These methods are as follows:

Method 1: Blob and Edge detection—Here we perform a scan over the fan blade and identify individual blobs of material that match or go over a threshold (RGB value). The reasoning for this is to capture areas of material which are near or past the threshold for a subsequent grinding operation.

Method 2: Region histogram and transition data—In this approach, the individual histogram data (Grayscale and RGB) of regions are obtained and tracked. This allows our system to observe when majority of our pixels, i.e., material color, has reached a particular threshold. Reaching the threshold indicates that a grinding operation is not required in that region. 

Method 3: Color Masking—This is a hybrid of the first two methods, color masking lets us do a wider scan of the blob / edge detection approach and mask out all values which are either above or below our threshold. This allows our system to observe how many pixels or percentage of material within a region of interest are above a threshold value. This region is then subsequently flagged for continual or a halt to the grinding process.

### 2.3. Particle Generator

Using the output of the Markovian chain in Section 2.2, regions of the digitized fan-blade image are assigned values. A particle representation of the fan blade is then constructed. Towards this, we assume that the fan-blade surface ∑ can be defined by Equation Z = f(X,Y) where *X* $\epsilon \;\{x_{1}$, $x_{2}$, $x_{3}$, … $x_{N}\}$, *Y* $\epsilon \;\{y_{1}$, $y_{2}$, $y_{3}$, … $y_{N}\}$ and *Z* $\epsilon \;\{z_{1}$, $z_{2}$, $z_{3}$, … $z_{N}\}$ are Cartesian coordinates of points *P* $\epsilon \;\{p_{1}$, $p_{2}$, $p_{3}$, … $p_{N}\}$, lying on the surface ∑. As a result, the surface ∑ is a collection of *P* $\epsilon \;\{p_{1}$, $p_{2}$, $p_{3}$, … $p_{N}\}$. From this collection, unit vectors $v_{p_{i}}$ normal to each $p_{i}$ on the fan-blade’s surface can be constructed if needed. To digitize the surface ∑ of the fan blade, we assume that there is a unit vector $v_{p_{i}}$ normal to each $p_{i}$ and hence $z_{i}$ on the fan blade. 

Using the RGB-D sensor, we assume that a picture taken of the surface ∑ is defined by I(χ)=∫h(χ, ϕ) r(χ)dχ where we condense *(X,Y)* into χ; r(χ) is a term associated with the radiance on the surface (in our case, we associated the radiance with color as defined by the Markovian-based surface region processor in Section 2.2); we represent the RGB-D sensor model as h(χ, ϕ) where $h\left(\chi ,\;\phi \right)=\;\frac{1}{\phi \sqrt{2\pi }}\;e^{-\frac{1}{2}x^{2}}$. In this work, we used ϕ=1 thereby resulting in the use of a standard normal distribution to model the RGB-D sensor. 

We derived virtual particles $vp_{i}$ for each detected point $p_{i}$ according to αI(χ) where α is a user defined scaling factor. The above results in a one-to-one mapping and provides a representation of the surface characteristics of the fan blade. During grinding, areas that are yet to be grinded stand out from areas that have been ground. As a result, the progress of grinding on the surface can be tracked overtime and subsequent algorithms used to plan grinding regimes to ensure uniform grinding coverage.

### 2.4. Digital Twin

To ensure that surface operations during the fan-blade reconditioning are done right the first time, we propose the use of a digital twin. In developing the digital twin, several information and knowledge were embedded into it as follows:

#### 2.4.1. Constraints of the Grinding Process

The first two layers of the fan blade must be removed so that they can be reapplied during reconditioning. When the second layer is removed, it has been done in such a way that there is enough surface roughness on the composite layer for the reapplied primer to bind. The test for surface roughness is done in terms of checking if water can bead onto the surface. This is because if the water does not bead, it is too smooth for the next coat of paint to apply. As a result, surface roughness is important.

Furthermore, during the material removal process, the grinding process should not heat the substrate to an extent that it gives off steam when water is applied. If the boiling point of water is exceeded, it will cause material deformation, surface deformation, and potentially cracks in the structure of the fan blade. Also, this will lead to material residual stresses. These stresses are caused by temperature gradients within the fan blade during grinding.

Finally, the grinding process must not injure or damage the inner most composite layer. Currently, if this layer is damaged, the fan blade is discarded leading to financial losses. As a result, getting this part of the process right is very crucial. Adhering to all these constraints while manually grinding puts a lot of strain on the operative, opens the possibility to errors and inconsistencies in the outcome of the process. Bearing the above constraints in mind, we ask how we can incorporate these constraints into grinding mathematical models for automation. 

#### 2.4.2. Deriving Grinding Parameters for Fan-Blade Reconditioning

To maintain the constraints presented in Section 2.4.1 and to derive the grinding process requirement for various layers, an understanding of the relationship between the process parameters and process measurements is needed. Furthermore, each layer in the fan blade has different properties and as such requires different process parameters to achieve certain surface outcomes.

In grinding, a rotating grinding wheel with an abrasive material is used. To derive the process parameters for grinding and the forces to be supplied to the robot’s end-effector, an understanding of the abrasive element and how it interacts with the material being ground is required. Typically, the abrasive material on the grinding wheel has randomly positioned abrasive grains. When used on grinding pads, the surface speeds at the wheel could reach speeds of 20–30 m/s and 150 m/s in high speed grinding [28,29]. 

**Figure 5:**
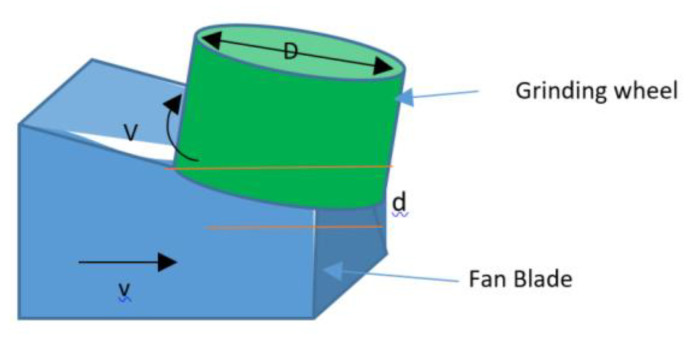
Grinding depiction picture.

In this work, a surface grinding operation was used for fan-blade reconditioning. Figure 5 gives a depiction of the surface grinding process where *t* is the undeformed chip thickness, *D* is the diameter of the grinding pad, *d* is the wheel depth of the cut, *V* is the velocity, *v* is the workpiece speed in relation to the direction of the grinding head, *l* is the undeformed length of the cut chip [28,29].

From the above parameters, the undeformed chip thickness *t* can be given by Equation (1). Where *C* is the number of cutting points per unit area of the wheel peripheral, *r* is the ratio of chip width to average undeformed chip thickness [28,29].

(1) $$t=\sqrt{\left(\frac{4v}{VCr}\right)\sqrt{\left(\frac{d}{D}\right)}}$$

With Equation (1), the thickness of material, *t* removed at each layer can be calculated and used to ensure that grinding is confined to a particular layer. For example, this will ensure that the fan-blade composite layer is not damaged during grinding. This variable was also useful in modelling and understanding the rate of transition through the material layers. 

Linked to the chip thickness variable is the material removal rate (*MRR*) given by Equation (2), where *w* is the cut width. $S_{M}$ is the strength of the material being grounded. In this work, $S_{M}$ varies from layer to layer due to the different constituents of each layer. The *MRR* variable was useful in obtaining an indication of how quickly the grinding process is commencing.

(2) $$MRR=\frac{dwv}{S_{M}}$$

To achieve a particular *MRR*, a power $P_{1}$ was required and this was calculated using Equation (3), where τ is a constant known as the specific energy. This constant will vary from layer to layer due to the different materials used in them.

(3) $$P_{1}=\tau MRR$$

Furthermore, power requirements, $P_{2}$, can also be calculated using the process parameters of the rotational speed of the grinding wheel ω, diameter of the wheel *D*, cutting Force *F_c_*, and rpm value *N* of the wheel as in Equations (4)–(6). These Equations were useful in understanding how to set the process parameters to achieve a particular MRR.

(4) $$P_{2}=\gamma \omega$$

(5) $$\gamma =F_{c}\frac{D}{2}$$

(6) ω=2πN

As discussed in the constraints section, temperature is an issue that needs to be kept constrained to a particular value to avoid problems associated with thermal effects generated during grinding. In this work, we use Equation (7) to estimate the surface temperature rise during a grinding operation. Equation (7), shows that the heat generated during grinding increases with depth of cut, *d*, wheel diameter, *D*, wheel speed, *V*, and reduces with increasing workpiece speed, *v*. Also, the depth of cut, *d*, makes the greatest contribution to the heat generated. As a result, it was not sufficient to use a deep depth of cut, *d*, to achieve a high *MRR* without risking damaging the material due to high temperature rises.

(7) $$T_{t}\;=T_{t-1}+\;D^{1/4}d^{3/4}{\left(\frac{V}{v}\right)}^{1/2}$$

To avoid temperature rises (> 90C) which could lead to distortions in the part, the grinding forces should be kept within range. The grinding forces are made up of the force normal to the work piece surface (thrust force $F_{N}$) and the force tangential to the wheel (cutting force, $F_{C}$). 

During cutting, the abrasive grains experience forces dictated by Equation (8).

(8) $$F_{C}=\left(\frac{v}{V}\sqrt{\left(\frac{d}{D}\right)}\right)S_{M}$$

From the above, the challenge is achieving an optimal balance between MRR while maintaining temperature (*T*) within safe limits to avoid thermal related problems [30]. 

Due to the nature of the problem, trial and error on real-world samples will be time consuming and subject to health and safety in terms of the hazardous environment and the robot control algorithms. As a result, we propose a physics-based particle model that enables us to exhaustively test out different grinding regimes and obtain their effects on surface quality and finish. 

#### 2.4.3. Physics-Based Particle Model

**Figure 6:**
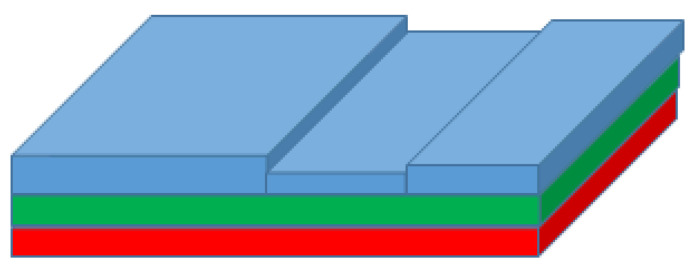
Geometry of simulated part being grounded.

As mentioned previously, the fan blade has many layers. However, the three topmost layers were the layers of interest. We depicted these layers in the digital twin with colors: Red (composite layer), Green (primer layer) and Blue (polyurethene layer) (See Figure 6).

It was assumed that each layer can be modelled using particles. This is because objects are made up particles that are bound together by forces. In this work, we assumed that the objects are held together by frictional forces at the interface of interaction (Equation (9)).

(9) $$F_{f}=\;\mu_{s}F_{n}$$

Where the friction value $F_{f}$ resists the tangential velocity of the grinding wheel’s velocity, *V*, $\mu_{s}$ is the coefficient of static friction and $F_{n}$ is the force normal to the surface of the object. We assume that $F_{f}$ determines the strength of the simulated part, $S_{M}$ as discussed in Equation (8). The abrasive grade, *C*, of the grinding wheel was simulated by using friction on the wheel. It was assumed that the higher the friction at the interface of the wheel and surface, the greater the potential for the wheel to overcome the particle to particle frictional force $F_{f}$. When the force *F* generated by both the grinding wheel’s tangential velocity and *C* exceeds the effects of the coefficient of static friction (Equation (10)), a particle from the part is removed.

(10) $$Fcos\left(\theta \right)>\;\mu_{s}\;Fsin\left(\theta \right)$$

**Figure 7:**
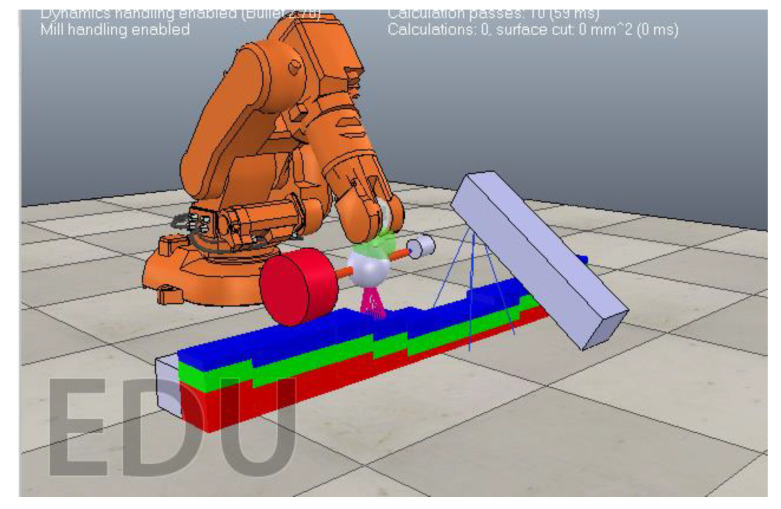
Digital twin particle-based simulation environment consisting of a robot arm. The robot arm is equipped with a rotating end-effector and a depth sensor for sensing the curvature of the digitized fan blade. The blue rectangle box depicts a mobile camera stand that captures the condition of the surface of the fan blade as it is treated.

An understanding of the above was used in the development of the physic-based particle model simulator in CoppeliaSim. To aid with experiments in the simulator, we categorized the variables in this work into 3 classes, input process variables; process measurements; surface outcomes as shown in Table 1. The simulation environment is shown in Figure 7 and comprised of a mobile camera (blue rectangle box), the digitized fan blade to be treated and a robot arm with an end-effector. The end-effector had a rotating grinding head and a depth sensor for sensing the curvature of the blade.

#### 2.4.4. Automated Grinding Control Algorithm

The RGB-D sensor (blue box in Figure 7) was used to collect data on depth and hence geometry of the part. This was necessary to ensure that a desired cutting force is maintained throughout the grinding operation. In simulations, the sensor was separated from the grinding end-effector by a longitudinal distance of 50 cm to prevent the dust from interfering with readings during the physical grinding process.

As a result, readings of the contour of the propeller (depth) at position *(x, y)* as well as the status of the surface of the propeller were obtained in advance of grinding as data item *(x, y, z, r, g, b).* A lagged memory framework was used to collect and store data in advance of the grinding end-effector. The memory lag network had five elements meaning that the sensor was five steps ahead of the grinding end-effector.

We use the knowledge embedded in Equation (7) to advance the grinding head so that the relative speed to the part, *v*, was sufficient to not exceed the temperature generated during grinding. Using Equation (8), we regulate the cutting force by keeping the rest of the process parameters constant and changing the depth of cut, *d*. This enabled us to meet the power constraints of the grinding motor head.

Due to process vibrations and other exogenous noise, there is bound to be errors in sensor readings and misalignments. As a result, the depth sensor readings were used to get within proximity of the geometry of the required cutting area while a compliant head design enabled us to compensate for errors arising from noisy depth readings.

**Figure 8:**
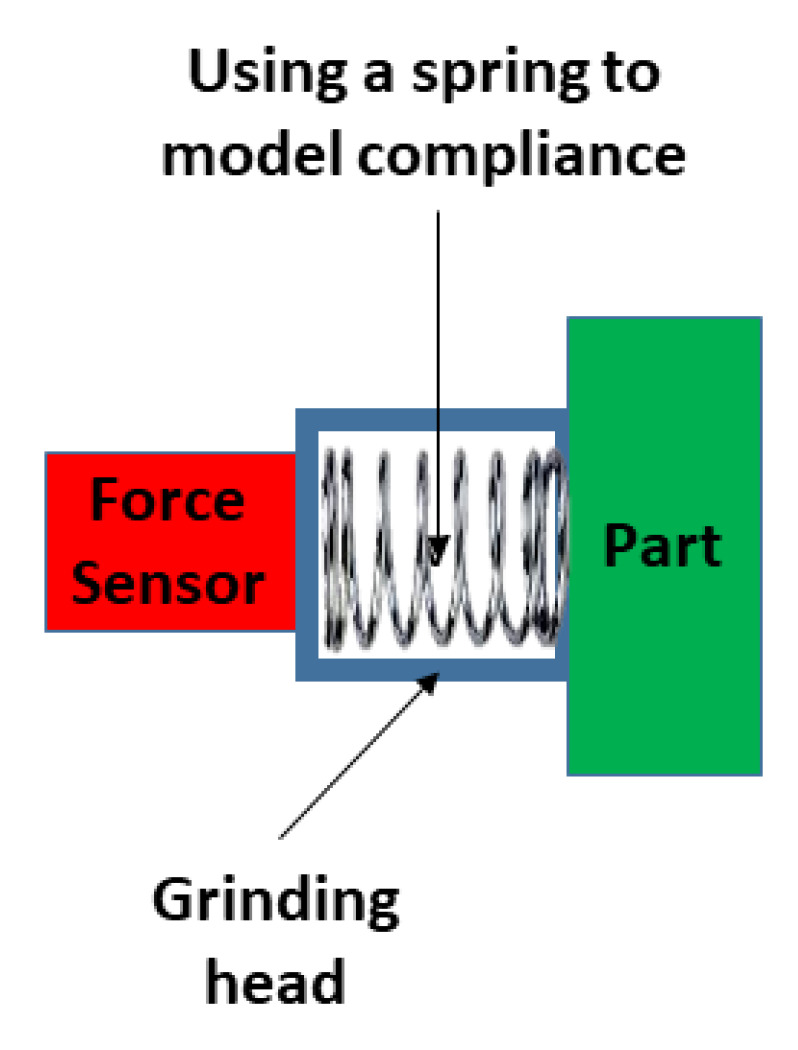
Spring system of the compliant head.

The compliant head was modelled as a spring system (Figure 8; Equation (11)) where the control algorithm had to maintain a certain force $F_{CR}$ and hence a desired depth of cut, *d*, throughout the grinding operation.

(11) $$F_{CR}=\;-kd$$

This is related to the cutting force in Equation (8). The required depth of cut, *d*, determines the amount of force that the end-effector was exerting on the surface of the part being ground. The greater the depth of cut, the greater the cutting force. In Equation (11), *k* is the spring constant. 

#### 2.4.5. Iterative Search Algorithm

Using the mathematical Equations from Section 2.4.2 and Section 2.4.4, we created an exhaustive search algorithm that iteratively searches for the best depth value, *d*, to apply to the fan-blade surface. This is because according to Equations (7) and (8), depth has a contribution to both the temperature rises during cutting as well as the grinding forces.

Using a range of depth values, we iteratively increase the depth value from −0.1 cm (just above the surface) to 0.5 cm. The value of −0.1 cm was trailed because of the compliant nature of the rotating end-effector as well as the sensor noise that is present in our depth sensors. Knowledge embedded in the Markovian chain (Figure 4) enabled us to assess the effects of the depth value on the fan-blade surface using simulated vision. Also, the MRR (Equation (2)) was used to assess the effects of the grinding forces on the fan blade.

In the future, we plan to make use of the vision-based Markovian chain and the MRR in more sophisticated heuristics to search for more optimal grinding parameters. Nevertheless, our current approach enabled us to assess the quality of the results produced by the digital twin. This was achieved by comparing the results of the digital twin with the results of the physical experiment.

## 3. Experiments and Results

In this section, we discuss the experimental set up and the result of experiments conducted. Physical experiments were first conducted. The results were then used to validate the Markovian-based surface region processor, particle generator, and the digital twin modules in Figure 3.

### 3.1. Physical Experiments 

**Figure 9:**
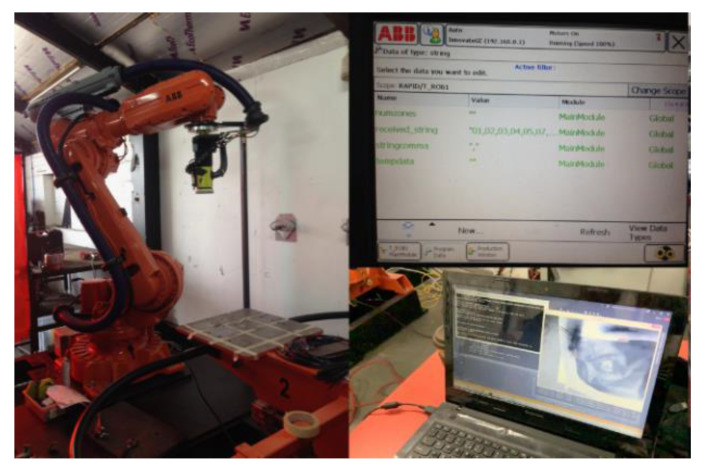
Main components of reconditioning cell during integration testing.

**Figure 10:**
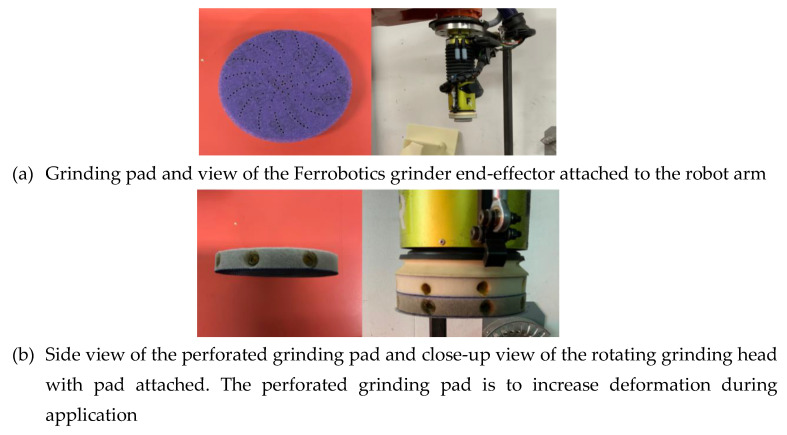
Robotic arm grinding head end-effector.

The setup used in the physical experiments was a robotic arm with a rotating head grinder and a PC as shown in Figure 9. Using Figure 8, a compliant rotating grinder head was incorporated into the setup (Figure 10).

**Figure 11:**
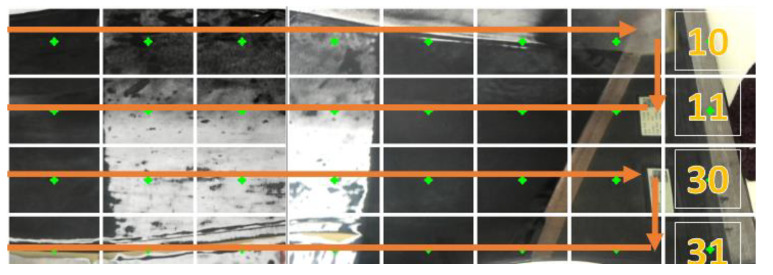
Fan-blade regions of interest for concave side.

**Figure 12:**
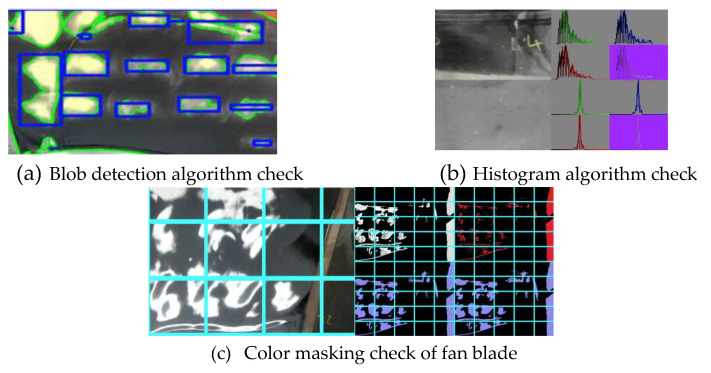
Visual inspection using the three proposed computer vision algorithms. The histograms in Figure 13b are used to check when the material color has reached a threshold. This is used to check if grinding is required or no longer required at different regions on the fan blade.

The first step in the physical experiments was to characterize the rotating head grinder to understand the rate of material removal and area coverage. The scanning process for both grinding and visual inspection followed a zig-zag approach as shown in Figure 11. The closed-loop system creates a scanned image of the full fan blade, performs image processing and then for each designated region of interest on the fan blade runs through each of the three visual methods (See Section 2.2 and Figure 12) to determine if they meet the criteria to stop grinding (Figure 2).

**Figure 13:**
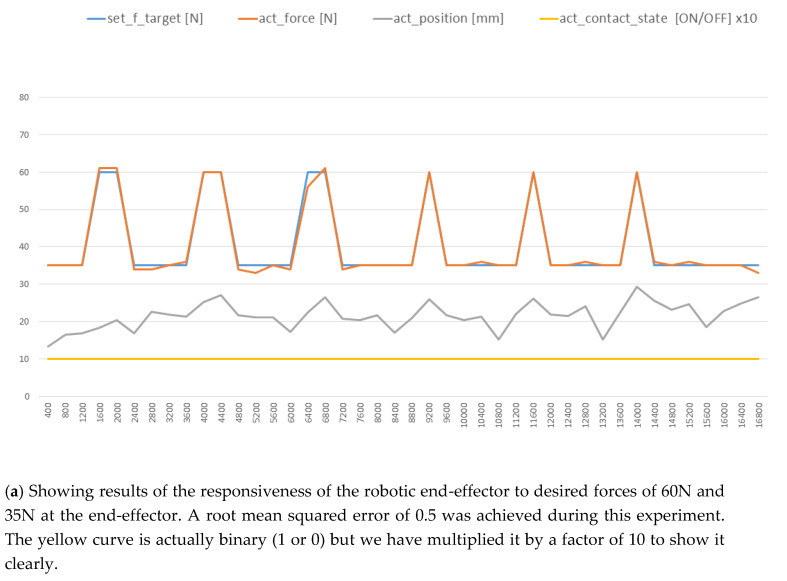

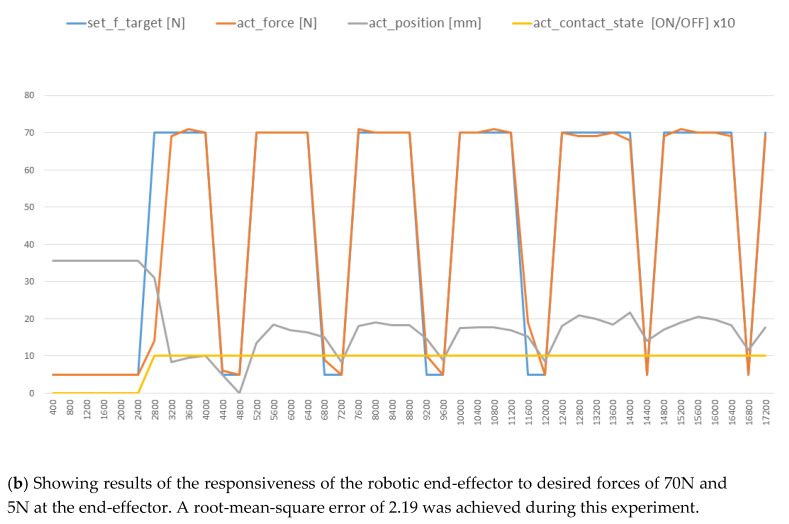
Validating the ability of the robotic end-effector to achieve various force commands in relation to a surface. The yellow curve is actually binary (1 or 0) but we have multiplied it by a factor of 10 to show it clearly.

**Figure 14:**
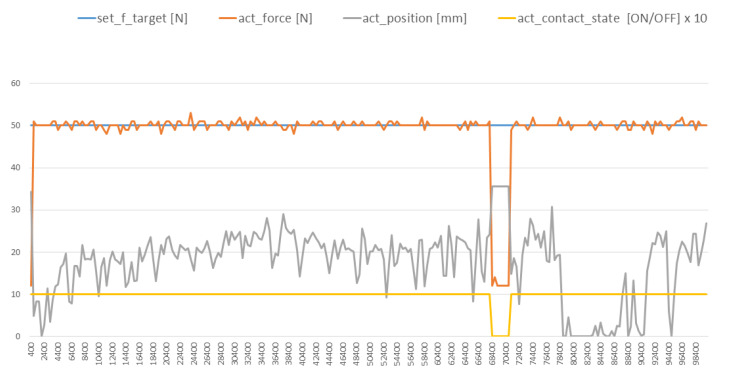
Graph showing the progress of the robotic end-effector during a reconditioning grinding operation. The curves show the target force (orange), actual force (blue) during grinding; actual height of the end-effector (grey) and whether there was contact or not contact with surface (yellow). The yellow curve is actually binary (1 or 0) but we have multiplied it by a factor of 10 to show it clearly.

We tested the ability of the robotic end-effector to follow various desired forces on the surface of the blade. This was achieved by controlling the depth of the robotic end-effector in relation to a surface. The force data from the embedded force sensor (see Figure 8) was used in the closed-loop feedback to achieve the appropriate depth of the robotic end-effector in respect to the fan-blade’s surface. Figure 13a,b show that the time lag between a new force being commanded and the robotic end-effector achieving it was less than 800 ms. A root-mean-square error of 0.5 was achieved in Figure 13a while a root-mean-square error of 2.19 was achieved in Figure 13b. During reconditioning grinding process on the fan blade, a constant desired force of 5 0N was applied throughout as shown by the blue curve in Figure 14. As a result of the aforementioned, the depth of the robot end-effector was controlled in relation to the curved surface of the fan blade. This made it possible to achieve a level of control over the generated surface temperature (Equation (7)) and the cutting forces (Equation (8)).

**Figure 15:**
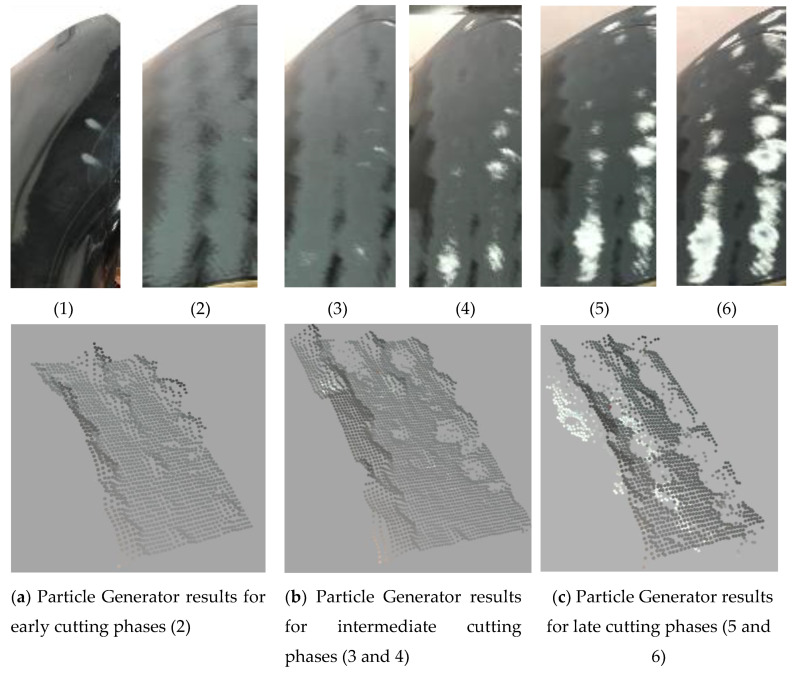
Progress of the proposed automated grinding cell. First row shows the effects of the grinding end-effector on the fan-blade surface while the second row shows the digitized surface using the particle generator (Section 2.3).

The grey curve in Figure 14, represents the height of the end-effector as it moves across the fan-blade surface. It can be seen that the curve follows the profile of the fan blade due to the use of both a combination of depth sensing, real-time force data, and a compliant end-effector. A root-mean-square error of 9.93 was achieved during this experiment. In support of this value, the roughness of the curves in Figure 14 are more pronounced than those in Figure 13. This could be because of the added load on the robotic end-effector’s controller when following the blade’s curvature. The yellow curve shows when there is contact with the surface of the fan blade and the end-effector. Between 60,000 ms and 80,000 ms, there was a time that the end-effector rose from the fan-blade surface to start another grinding round on the fan-blade surface. The progress of the automated grinding on a fan blade is shown in Figure 15. 

### 3.2. The Particle Model Results 

The results of the particle generator are shown in the second row of Figure 15. From Figure 15a–c, it will be seen that as the grinding progresses, the digitized surface of the fan blade progressed from a smooth finish to rough finish. These results show that the particle generator module (Section 2.3) could provide a digitized version of the fan-blade’s surface as grinding progresses. The results could then be used to determine which part of the fan blade needed more grinding and which did not. Furthermore, the digitization of the fan-blade surface into particle format enables the ability to generate region-based grinding while keeping in mind the compliant nature of the end-effector. 

### 3.3. Physics-Based Particle Model

**Figure 16:**
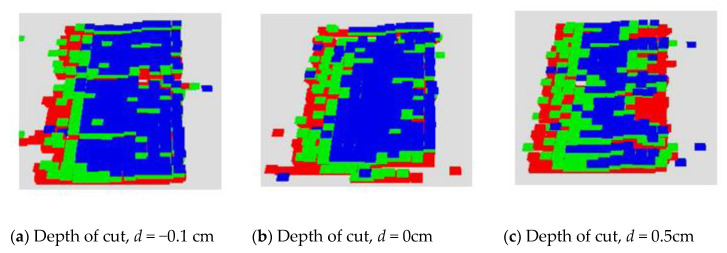
Composite images of the grinding results at different depth of cut values.

**Figure 17:**
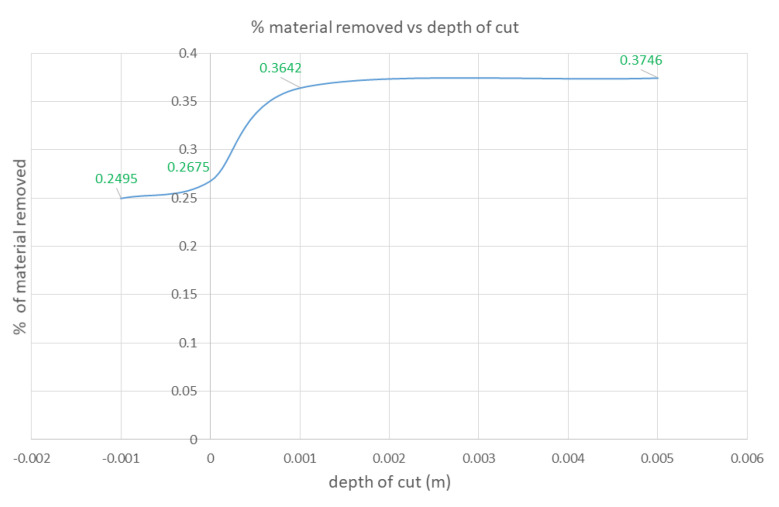
Results of increasing depth of cut on the amount of material removed.

The robot arm was moved at a constant speed, v of 5 cm/sec with the grinding head velocity, V of 50 rad/sec. Because CoppeliaSim is a physics-based simulator, it can simulate the effects of vibrations from the motor as well as grinding head compliance. Depth of cuts of −0.1cm, 0cm, 0.1cm and 0.5 cm were tested. The results obtained are shown in Figure 16 and Figure 17, where it can be seen that increasing depth of cut resulted in more material removal.

Nevertheless, as the depth of cut increases, Figure 17 shows that a level off is achieved in the MRR. This is because if a large depth of cut is required or commanded, the motor will be pressed too hard onto the surface. Due to the high pressure and high frictional force at the surface of the fan blade, the achievable velocity, *V*, of the rotating head will be constrained. This becomes more pronounced as a large depth of cut is required or commanded. In the physical world, this could lead to a motor burn out. As a result, our digital twin physic-based model was able to embed this reality as well. These results provided proof of its validity. Furthermore, if the velocity, *V*, were not kept in check, temperature rises would lead to fan-blade surface damages as discussed in Section 2.4.1 and Equation (7).

## 4. Discussion

In this paper, we explored how to automate material removal during fan-blade reconditioning process for high-value aerospace MRO. We presented an approach that makes use of a digital twin supported by a vision-based digitization scheme. The scheme enables surfaces of fan blades to be digitized into a particle representation for the purposes of testing various grinding regimes on the fan blade. In the digital twin, we embedded knowledge of the constraints to be adhered to during grinding. These constraints guided the search for optimal grinding parameters. Knowledge of the grinding parameters were used to derive the process requirements to achieve certain rates of material removal. The grinding parameters were also used to ensure that surface temperatures did not raise above values that could cause thermal deformation.

A physics-based particle model was built to simulate the layers in the fan-blade structure. Frictional forces were used to simulate the bonding forces between the particles. Choosing the frictional parameters of the particles in the fan blade were performed empirically. In future work, automatic characterization of the bonding forces between the particles in the fan blade would be a worthwhile research challenge. Nevertheless, the results produced by the physics-based particle model in the digital twin were comparable to the real-world results. For example, we were able to confirm that larger cut depths removed more material from the simulated fan-blade surface. This corresponded to the results observed visually and quantitatively on the physical fan-blade’s surface. We were also able to confirm that similarly to the physical automation cell, a large depth of cut command sent to the simulated robotic arm leads to large frictional forces at the fan-blade grinder interface. In the real world, this would lead to over loading of the motor. A similar effect was observed in the digital twin’s simulation.

Using iterative search algorithms, it was possible to experiment in the digital twin and find out the optimal grinding parameters. The digital twin enabled us to test various grinding regime hypotheses safely, cheaply, and quickly before implementing the optimal result on a physical robot arm.

The grinding parameters were derived using a mathematical understanding of the process (Equations (1) to (8)). The mathematical understanding of the process gave us insight into how switching from a standard 180 grit paper to a higher grit led to higher material removal (Equations (2) and (8)). Furthermore, it was discovered that the material removal was rather non-uniform, with specific areas of the fan blade being left untouched while others were ground away fairly quickly once the primer was revealed. This revealed that the material at various layers had different characteristics. This was modelled using Equation (3).

Through observation, it was discovered that the grinder head and the angle it was impacting the fan blade was having a somewhat uncontrollable effect on the material removal, leading to a non-uniform distribution of force. This was due to not considering the curved geometry of the fan blade. As a result, the control algorithm was developed so that the pressure applied to the center point of each targeted region was higher than the pressure applied to the outside of the grinding pad. Additionally, the grinding head was made compliant by adding some holes into the foam material shown in Figure 10a. The overall aim of these changes was to alleviate the pressure applied on the curvature of the blade. This led to more uniform distribution of material removal during each cycle.

Grinding the surface of the fan blade was achieved using a zig-zag motion across its surface (Figure 11). Using a compliant end-effector design, it was possible to follow the curvature of the fan-blade’s surface (Figure 14) and achieve automated grinding using a force of 50N. In order ensure an optimal grinding regime, we make use of a combination of a Markovian-based surface region processor algorithm and particle generator. The particle generator enabled us to produce a digitized version of the fan-blade’s surface (Figure 15).

Using an iterative search algorithm, we were able to develop preliminary algorithms that can search for optimal grinding parameters. In the future, more sophisticated algorithms will be researched. Nevertheless, our experience and findings from this research lead us to the conclusion that a closed-loop system for automating the material removal process is achievable. However, as seen in the first row of Figure 15, more work is needed to have finer control over the grinding end-effector. These results can be improved by better integration of the results of the particle generator module with the rest of the other modules in our proposed approach. The results of the physics-based particle model in Figure 16 and Figure 17 show that it is suitable for use in testing out various hypothetical grinding regimes. This will be impactful in ensuring that fan-blade reconditioning is done right the first time in MRO operations in the aerospace sector.

## 5. Conclusions

The UK is a major player in the domain of aerospace and the commercial air transport MRO market. The advent of industrial Internet of Things (IoT), digital manufacturing and current trend of integrating more autonomous or robotic solutions into this domain means that we can begin to investigate autonomous solutions that would not have been possible previously. Furthermore, the framework presented in this paper contributes to the body of knowledge that is exploring the use of automated grinding in other sectors such as Space Optics [31,32,33].

This paper sets out to automate a manual activity undertaken by human engineers within a major aerospace MRO facility; that of fan-blade reconditioning. The manual activity involved grinding away the surface of a fan blade. This activity produces a hazardous environment which puts the worker at risk. As a result, the worker is forced to wear protective equipment which impedes movements, leads to an increase in errors and hence increase in fan-blade scrappages.

In this paper, we proposed the design and development of an automation cell supported by low-cost vision-based sensing and a digital twin. The cell was capable of continuously tracking and removing the coating material of a fan blade in a closed-loop approach. Our initial tests and findings successfully demonstrated the applicability of automation to fan-blade reconditioning in the MRO engine process. Nevertheless, more work is needed to improve the overall outcomes.

The main challenge we faced when designing and building the automated reconditioning cell was cost. This is because majority of the companies involved in remanufacturing or refurbishing of aerospace fan blades are SMEs. As a result, the parts used in the automation cell had to be affordable to drive uptake and hence afford these SMEs an opportunity to improve their productivity. Consequently, the sensors and sensing strategies used for sensing the grinding progress during the reconditioning process had to meet a fine balance between effectiveness and cost. To achieve this, we used a Markovian-based strategy to support the low-cost sensing strategies we used.

Another challenge that was faced was the availability of parts for validation. This is because the fan blades are quite costly in themselves and would be expensive to test our reconditioning automation strategy on physical blades. As a result, a digital twin was developed and used to understand the grinding conditions specific to the grinding of composite fan blades. This enabled us to experiment and gain a knowledge of intricacies of the process in parallel with the physical development of the automation cell. This parallel development also enabled us to parameterize and validate the results our digital twin.

Due to the grinding environment, dust and dirt was another challenge that arose. Initially, this affected our sensor readings. However, using the lagged memory framework presented in the text and the digital twin, we were able to mitigate the effects of this. The lagged memory framework also provided some support for the complex curved geometry of fan blades. This enabled us to predict in advance the height to which to raise the robotic grinding end-effector. Nevertheless, the grinding head had to be adapted and made bespoke for it to be more compliant and follow the curvature of the blade. As a result, in the future, this would need to be better characterized so that the results could be fed back to manufacturers for mass production.

In the future, further refinement of the automated cell, vision-based algorithms, and digital twin software will be undertaken, with the aim to improve the overall material removal process while meeting constraints imposed by the fan-blade composite materials. When compared with manual grinding, the aim issue that constrains the performance of automated grinding is embedding the knowledge of a skill worker into an automated cell. This is a not trivial process as this manual skill was gained and honed over several years. Some of these skills are in fact tacit and sometimes difficult for the worker to verbalize. Finding a way to digitize and effectively transfer this skill to an automated cell impacts every module in our architecture. However, the use of digital twins and machine learning algorithms could provide a way of experimenting and exploring the tacit mechanisms developed by these workers over a period of years.

## Figures and Tables

**Table 1:** A table showing the input process parameters, process measures, and output parameters.

Classes.	Variables
**Input process variables**	1. Wheel speed *V* 2. Feed speed *v* 3. Depth of cut *d* 4. Wheel diameter D 5. Friction f or abrasive grade C 6. Strength of part $S_{M}$ 7. Cutting force $F_{c}$
**Measures**	1. MRR (Equation (2)) 2. Power requirements, *P* (Equation (3) to (6)) 3. Temperature, *T* (Equation (7)) 4. Grain force (Equation (8)).
**Output**	1. Residual stress/heat checking (Visual checks via cracks in the fan blade after each operation). 2. Surface roughness.
